# Supplementary figures and images for: Pneumolysin as a target for new therapies against pneumococcal infections: A systematic review
Source: PLoS One. 2023 Mar 22;18(3):e0282970. doi: 10.1371/journal.pone.0282970 (PMC10032530; doi:10.1371/journal.pone.0282970)

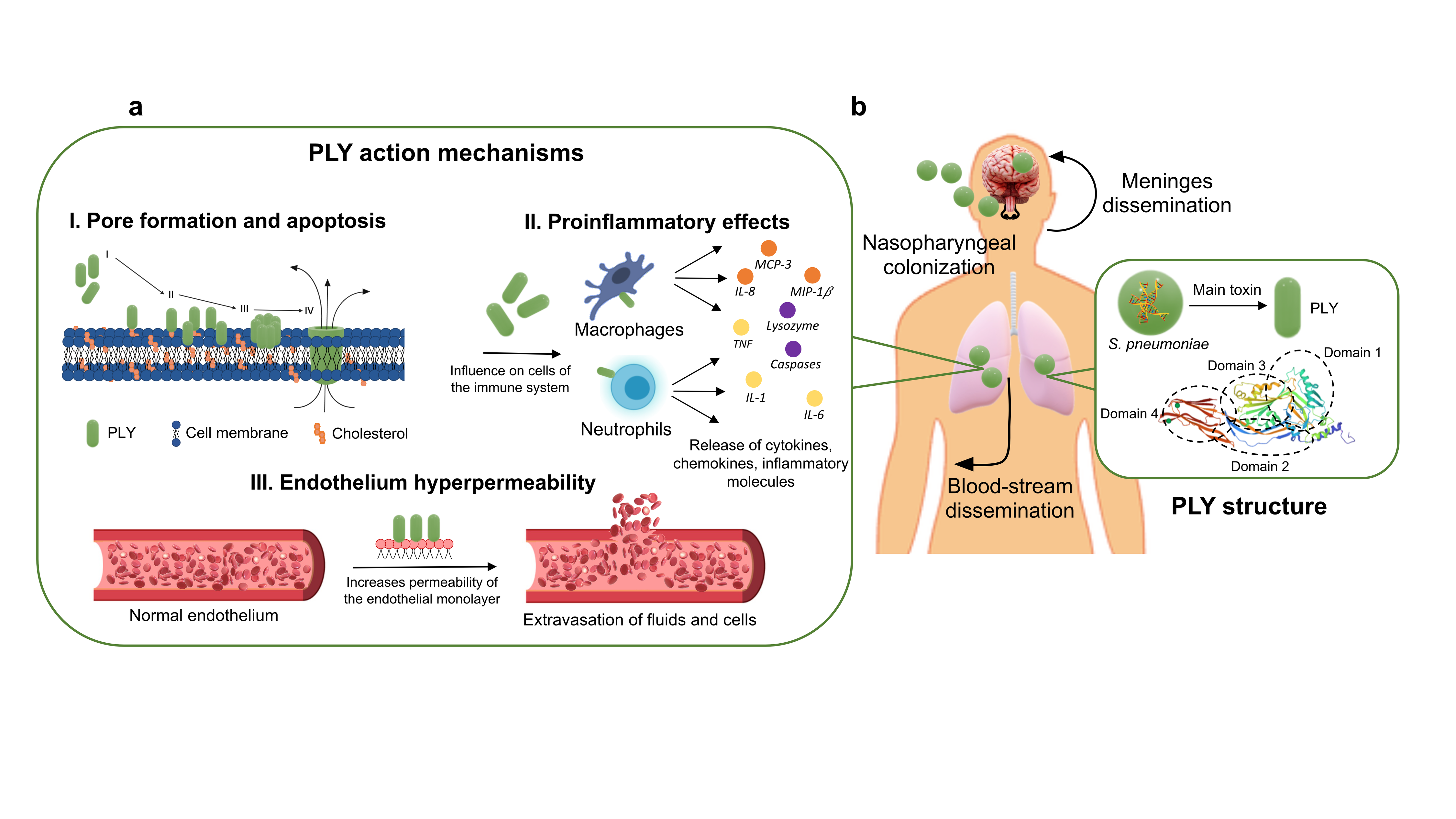

Supplement: S1 Fig — (a) Toxin interactions with cellular membranes (I), human immune system (II), and endothelium (III) are depicted. (b) The pneumococcus spreading routes and the main protein domains relevant to the structure of PLY are indicated. (TIF) [file pone.0282970.s001.tif]
