# Supplementary material for: Pneumolysin as a target for new therapies against pneumococcal infections: A systematic review
Source: PLoS One. 2023 Mar 22;18(3):e0282970. doi: 10.1371/journal.pone.0282970 (PMC10032530; doi:10.1371/journal.pone.0282970)
Supplement: S2 Table — (DOCX) [file pone.0282970.s003.docx]

**Table S2.** Data extraction items.

| **Study Identification Information** | Author, year | | Study Type (Animal, in vitro, both) | |
| --- | --- | --- | --- | --- |
|  | DOI | | Chemical Name/CAS | |
|  | URL | |  | |
|  | Funding Source | |  | |
| **In Vivo (Animal testing)** | | | | |
| SP strain | Animal Group Strain | | Experiment Type (pneumonia, sepsis, others) | |
| Sample size | Doses | | Dosing Regime | |
| +/- controls | Route | | Observation Duration (h) | |
| Statistical data |  | | | |
| Results Notes |  | | | |
| Additional information |  | | | |
| **In Vitro (Culture testing)** | | | | |
| SP strain | | MIC | | Observation Time |
| Cell Type | | Dose Units | | Cytotoxicity |
| Cell Tissue | | Dosing Regime | |  |
| Assay Type | |  | |  |
| Statistical data | |  | | |
| Results Notes | |  | | |
| Additional information | |  | | |

SP strain, *Streptococcus pneumoniae* strain; MIC, Minimum inhibitory concentration.
