# Supplementary material for: Pneumolysin as a target for new therapies against pneumococcal infections: A systematic review
Source: PLoS One. 2023 Mar 22;18(3):e0282970. doi: 10.1371/journal.pone.0282970 (PMC10032530; doi:10.1371/journal.pone.0282970)
Supplement: S3 Table — (DOCX) [file pone.0282970.s004.docx]

**Table S3.** Study quality assessment based on the OHAT Risk Assessment

| Author, Year | System | Selection Bias | | | Confounding Bias | Performance Bias | | Attrition/ Exclusion Bias | Detection Bias | | Risk of Bias |
| --- | --- | --- | --- | --- | --- | --- | --- | --- | --- | --- | --- |
|  |  | *Was administered dose or exposure level adequately randomized?* | *Was allocation to study groups adequately concealed?* | *Did selection of study participants result in appropriate comparison group?* | *Did the study design or analysis account for important confounding and modifying variables* | *Were experimental conditions identical across study groups?* | *Were the research personnel and human subjects blinded to the study group during the study?* | *Were outcome data complete without attrition or exclusion from analysis?* | *Can we be confident in the exposure characterization?* | *Can we be confident in the outcome assessment?* |  |
| Domon et al., 2021 | Animal | ****** | **-** | **NA** | ***** | ****** | **-** | ***** | ****** | ****** | **Probably Low** |
| Marquart et al., 2007 | Animal | ****** | **-** | **NA** | ***** | ****** | ****** | ***** | ****** | ****** | **Probably Low** |
| Majhi et al., 2014 | Animal | ****** | **-** | **NA** | ***** | ****** | ***** | ***** | ****** | ****** | **Probably Low** |
| Rosch et al., 2010 | Animal | ****** | **-** | **NA** | ***** | ****** | **-** | ***** | ****** | ****** | **Probably Low** |
| Lv et al., 2020 | Animal | ***** | **-** | **NA** | ***** | ****** | **-** | ***** | ****** | ****** | **Probably Low** |
| Xu et al., 2020 | Animal | ***** | **-** | **NA** | ***** | ***** | **-** | **-** | ***** | ***** | **Low** |
| Hupp et al., 2017 | Animal | ****** | **-** | **NA** | ****** | ****** | ****** | ***** | ****** | ****** | **Probably Low** |
| Song et al., 2017a | Animal | ****** | **-** | **NA** | ***** | ****** | **-** | ***** | ****** | ****** | **Probably Low** |
| Li et al., 2015 | Animal | ****** | **-** | **NA** | ****** | ****** | **-** | **+** | ****** | ****** | **Probably Low** |
| Song et al., 2016 | Animal | ***** | **-** | **NA** | ***** | ****** | **-** | ***** | ***** | ***** | **Low** |
| Zhao et al., 2016 | Animal | ***** | **-** | **NA** | ***** | ***** | **-** | ***** | ***** | ***** | **Low** |
| Le et al., 2015 | Animal | ****** | **-** | **NA** | ****** | ****** | ***** | ****** | ****** | ****** | **Probably Low** |
| Zhao et al., 2017c | Animal | ***** | **-** | **NA** | ***** | ****** | **-** | ***** | ***** | ***** | **Low** |
| Srivastava et al., 2005 | Animal | ****** | **-** | **NA** | ***** | ****** | **-** | ****** | ****** | ****** | **Probably Low** |
| Li et al., 2020 | Animal | ***** | **-** | **NA** | ***** | ***** | **-** | ***** | ***** | ***** | **Low** |
| Zhao et al., 2017a | Animal | ***** | **-** | **NA** | ***** | ***** | **-** | ***** | ***** | ***** | **Low** |
| Zhao et al.,2017b | Animal | ***** | **-** | **NA** | ***** | ***** | **-** | ***** | ***** | ***** | **Low** |
| Subramanian et al., 2020 | Animal | ****** | **-** | **NA** | ***** | ****** | ***** | ****** | ****** | ****** | **Probably Low** |
| Gutbier et al., 2017 | Animal | ****** | **-** | **NA** | ***** | ****** | **-** | ***** | ****** | ****** | **Probably Low** |
| Guo et al., 2021 | Animal | ***** | **-** | **NA** | ***** | ***** | **-** | ***** | ***** | ***** | **Low** |
| Green et al., 2008 | Animal | ***** | **-** | **NA** | ***** | ****** | ***** | ****** | ****** | ****** | **Probably Low** |
| García-Suárez et al., 2004 | Animal | ****** | **-** | **NA** | ***** | ****** | **-** | ****** | ****** | ****** | **Probably Low** |
| Kaur et al., 2014 | Animal | ****** | **-** | **NA** | ***** | ****** | **-** | ****** | ****** | ****** | **Probably Low** |
| Witzenrath et al., 2009 | Animal | ****** | **-** | **NA** | ***** | ****** | ***** | ****** | ****** | ****** | **Probably Low** |
| Statt et al., 2015b | Animal | ****** | **-** | **NA** | ***** | ****** | **-** | ****** | ****** | ****** | **Probably Low** |
| Shigematsu et al., 2016 | Animal | ****** | **-** | **NA** | ***** | ****** | **-** | ***** | ****** | ****** | **Probably Low** |
| Lucas et al., 2012b | Animal | ****** | **-** | **NA** | ***** | ****** | **-** | ***** | ****** | ****** | **Probably Low** |
| Lucas et al., 2012a | Animal | ****** | **-** | **NA** | ***** | ****** | **-** | ***** | ****** | ****** | **Probably Low** |
| Chang et al., 2020 | Animal | ***** | **-** | **NA** | ***** | ****** | **-** | ***** | ***** | ***** | **Low** |
| Franco-Vidal et al., 2008 | Cellular | **NA** | **-** | **NA** | ***** | ** | **-** | ***** | ***** | ****** | **Low** |
| Wippel et al., 2011 | Cellular | **NA** | **-** | **NA** | ****** | ** | **-** | ***** | ****** | ****** | **Probably Low** |
| Nerlich et al., 2021 | Cellular | **NA** | **-** | **NA** | ****** | ** | **-** | ***** | ****** | ****** | **Probably Low** |
| Fickl et al., 2005 | Cellular | **NA** | **-** | **NA** | ***** | ** | **-** | ***** | ****** | ****** | **Probably Low** |
| Qi et al., 2020 | Cellular | **NA** | **-** | **NA** | ***** | * | **-** | ***** | ****** | ****** | **Probably Low** |
| Maatsola et al., 2020 | Cellular | **NA** | **-** | **NA** | ***** | * | **-** | ***** | ****** | ****** | **Probably Low** |
| Song et al., 2017b | Cellular | **NA** | **-** | **NA** | ***** | * | **-** | ***** | ****** | ****** | **Probably Low** |
| Ding et al., 2021 | Cellular | **NA** | **-** | **NA** | ***** | * | **-** | ***** | ***** | ***** | **Low** |
| Lv et al., 2021 | Cellular | **NA** | **-** | **NA** | ***** | * | **-** | ***** | ***** | ***** | **Low** |
| N'Guessan et al., 2005 | Cellular | **NA** | **-** | **NA** | ***** | * | **-** | ***** | ****** | ****** | **Probably Low** |
| Zhou et al., 2020 | Cellular | **NA** | **-** | **NA** | ***** | * | **-** | ***** | ***** | ***** | **Low** |
| Oiteno et al., 2021 | Cellular | **NA** | **-** | **NA** | ***** | * | **-** | ***** | ****** | ****** | **Probably Low** |

** Definitely Low; * Probably Low; - Probably High or not information; -- Definitely High; NA, not applicable.
