# Supplementary material for: Pneumolysin as a target for new therapies against pneumococcal infections: A systematic review
Source: PLoS One. 2023 Mar 22;18(3):e0282970. doi: 10.1371/journal.pone.0282970 (PMC10032530; doi:10.1371/journal.pone.0282970)
Supplement: S4 Table — (DOCX) [file pone.0282970.s005.docx]

**Table S4.** Articles and number of clinical trials.

| **Authors, Year** | **DOI** | **County** | **Molecule** | **Clinical Trials (global)** | **Clinical Trials (pneumonia)** | **Clinical Trials (sepsis)** |
| --- | --- | --- | --- | --- | --- | --- |
| Chang et al., 2020 | 10.3390/cells9051183 | Taiwan | C70PLY | 0 | 0 | 0 |
| Ding et al., 2021 | 10.1016/j.micinf.2021.104888 | China | Hederagenin | 0 | 0 | 0 |
| Domon et al., 2021 | 10.1128/Spectrum.00318-21 | Japan | Clarithromycin | 1894 | 138 | 19 |
| Fickl et al., 2005 | 10.1111/j.1365-2249.2005.02757.x | South Africa | Docosahexaenoic acid | 2096 | 8 | 15 |
| Franco-Vidal et al., 2008 | 10.1159/000108763 | France | Zinc | 3580 | 64 | 23 |
| García-Suárez et al., 2004 | 10.1128/IAI.72.8.4534-4540.2004 | Spain | MAb antibodies | 0 | 0 | 0 |
| Green et al., 2008 | 10.1167/iovs.07-0492 | USA | Polyclonal antiserum | 0 | 0 | 0 |
| Guo et al., 2021 | 10.1016/j.jep.2021.114133 | China | MXSGT | 0 | 0 | 0 |
| Gutbier et al., 2017 | 10.1186/s13054-017-1851-6 | Germany | Vasculotide | 0 | 0 | 0 |
| Hupp et al., 2017 | 10.1111/bph.14027 | Switzerland | Magnesium | 3807 | 23 | 21 |
| Kaur et al., 2014 | 10.1128/IAI.02124-14 | USA | Human antibodies | 0 | 0 | 0 |
| Le et al., 2015 | 10.1038/srep11886 | Malaysia | DM3 peptide | 4 | 0 | 0 |
| Li et al., 2015 | 10.1038/srep17668 | China | β-sitosterol | 132 | 0 | 0 |
| Li et al., 2020 | 10.1111/jphp.13279 | China | Acacetin | 1 | 0 | 0 |
| Lucas et al., 2012a | 10.1073/pnas.1121075109 | USA | JI-34 | 0 | 0 | 0 |
| Lucas et al., 2012b | 10.1165/rcmb.2011-0332OC | USA | TIP peptide | 3 | 1 | 0 |
| Lucas et al., 2012b | 10.1165/rcmb.2011-0332OC | USA | Ro 32-0432 | 0 | 0 | 0 |
| Lv et al., 2020 | 10.1016/j.micpath.2019.103934 | China | Quercetin | 293 | 5 | 0 |
| Lv et al., 2021 | 10.1016/j.micpath.2020.104683 | China | Dryocrassin | 0 | 0 | 0 |
| Maatsola et al., 2020 | 10.3390/antibiotics9120930 | Finland | Pentagalloylglucose | 1 | 0 | 0 |
| Majhi et al., 2014 | 10.1128/AAC.03245-14 | India | Ceftriaxone + Levofloxacin | 29 | 22 | 0 |
| Marquart et al., 2007 | 10.1167/iovs.07-0017 | USA | Cholesterol | 24000 | 30 | 42 |
| Oiteno et al., 2021 | 10.4014/jmb.2105.05024 | China | Aloe-emodin | 4 | 0 | 0 |
| Qi et al., 2020 | 10.1111/jam.14769 | China | Betulin | 13 | 0 | 0 |
| Rosch et al., 2010 | 10.1172/JCI39843 | USA | Simvastatin | 1921 | 9 | 13 |
| Shigematsu et al., 2016 | 10.1038/srep34560 | Japan | Montelukast | 618 | 5 | 0 |
| Song et al., 2016 | 10.1016/j.fitote.2016.09.017 | China | Apigenin | 21 | 0 | 0 |
| Song et al., 2017 | 10.1111/jcmm.13179 | China | Epigallocatechin gallate | 196 | 0 | 0 |
| Song et al., 2017 | 10.1007/s10482-017-0880-0 | China | Juglone | 0 | 0 | 0 |
| Srivastava et al., 2005 | 10.1128/IAI.73.10.6479-6487.2005 | USA | zVAD | 1 | 0 | 0 |
| Subramanian et al., 2020 | 10.15252/emmm.202012695 | Sweden | MRC‐1 peptides | 0 | 0 | 0 |
| Wippel et al., 2011 | 10.1093/infdis/jir434 | Germany | Calcium | 19152 | 36 | 52 |
| Witzenrath et al., 2009 | 10.1097/CCM.0b013e3181959814 | Germany | PDP | 0 | 0 | 0 |
| Xu et al., 2020 | 10.1248/bpb.b20-00034 | China | Ephedrine hydrochloride | 1020 | 2 | 0 |
| Zhao et al., 2016 | 10.1124/mol.115.100610 | China | Verbascoside | 18 | 0 | 0 |
| Zhao et al., 2017a | 10.1248/bpb.b16-00598 | China | Amentoflavone | 1 | 0 | 0 |
| Zhao et al., 2017c | 10.1016/j.lfs.2017.04.002 | China | Shikonin | 2 | 0 | 0 |
| Zhao et al.,2017b | 10.1248/cpb.c16-00999 | China | Morin | 431 | 0 | 0 |
| Zhou et al., 2020 | 10.1021/acsomega.0c00460 | China | Oleanolic Acid | 33 | 0 | 1 |
