# Supplementary material for: Pneumolysin as a target for new therapies against pneumococcal infections: A systematic review
Source: PLoS One. 2023 Mar 22;18(3):e0282970. doi: 10.1371/journal.pone.0282970 (PMC10032530; doi:10.1371/journal.pone.0282970)
Supplement: S5 Table — (DOCX) [file pone.0282970.s006.docx]

**Table S5.** Chemical structures of molecules with effects against PLY.

| **Molecule (No CAS)** | **Structures** | **Molecule type** |
| --- | --- | --- |
| Acacetin (480-44-4) |  | Flavonoid, polyphenol (natural) |
| Amentoflavone (1617-53-4) |  | Flavonoid, polyphenol (natural) |
| Morin (480-16-0) |  | Flavonoid, polyphenol (natural) |
| Apigenin (520-36-5) |  | Flavonoid, polyphenol (natural) |
| Epigallocatechin gallate  (989-51-5) |  | Flavonoid, Polyphenol (natural) |
| Quercetin (117-39-5) |  | Flavonoid |
| Dryocrassin ABBA (12777-70-7) |  | Flavonoid |
| Verbascoside (61276-17-3) |  | Phenylpropanoid glycoside, Polyphenol  (natural) |
| Pentagalloylglucose (14937-32-7) and gemin A (82220-61-9) |  | Tannins, polyphenols (natural) |
| Juglone (481-39-0) |  | Naphthoquinone |
| Shikonin (517-89-5) |  | Naphthoquinone (natural) |
| Aloe-emodin (481-72-1) |  | Anthraquinone (natural) |
| Ephedrine hydrochloride  (50-98-6) |  | Phenethylamines (natural) |
| Pseudoephedrine hydrochloride (345-78-8) |  | Phenethylamines (natural) |
| Ephedrine hydrochloride  (50-98-6) |  | Phenethylamines (natural) |
| Pseudoephedrine hydrochloride (345-78-8) |  | Phenethylamines (natural) |
| Methylephedrine (552-79-4) |  | Phenethylamines (natural) |
| Amygdalin (29883-15-6) |  | Amygdalin and Glycyrrhetinic acid |
| Prunasin (99-18-3) |  | cyanogenic glycoside |
| Glycyrrhetinic acid (471-53-4) |  | Glycyrrhetinic acid |
| Hederagenin (465-99-6) |  | Triterpenoid |
| Betulin (473-98-3) |  | Triterpene |
| Oleanolic Acid (508-02-1) |  | Pentacyclic triterpenoids (natural) |
| Cholesterol (57-88-5) |  | Sterol (natural) |
| β-sitosterol (83-46-5) |  | Sterol (natural) |
| Simvastatin (79902-63-9) |  | Statins (Synthetic) |
| Simvastatin (79902-63-9) |  | Statins (Synthetic) |
| Docosahexaenoic acid  (6217-54-5) |  | Fatty acids Omega3 (natural) |
| 9-(6-phenyl-2-oxohex-3-yl)-2-(3,4-dimethoxybenzyl)-purin-6one (190666-14-9) |  | Purin-6-ones (Synthetic) |
| CysLT1 antagonists Montelukast (158966-92-8) |  | Drug (Synthetic) |
| Clarithromycin (81103-11-9) |  | Antibiotics – Macrolide (Synthetic) |
| Ceftriaxone (104376-79-6) |  | Antibiotics – Cephalosporin (Synthetic) |
| Levofloxacin (100986-85-4) |  | Antibiotics – Quinolone (Synthetic) |
| C-terminal 70 amino acids of PLY (C70PLY) |  | Peptide |
| DM3 peptide: GLFDIWKWWRWRR-NH2  Indolicidin peptide derivative: ILAWKWAWWAWRR-NH2 |  | Synthetic hybrid peptides |
| Mannose receptor peptides (MRC-1) |  | Peptide |
| Bisindolylmaleimide XI hydrochloride (145333-02-4) |  | Peptides and drug (Synthetic) |
| GHRH agonist: JI-34 peptide |  | Peptide analogs |
| Vasculotide | 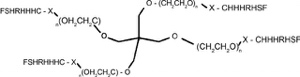 | Peptide modified |
| Z-VAL-ALA-OH (24787-89-1) |  | Peptide modified |
| zVAD-fmk (187389-52-2) | 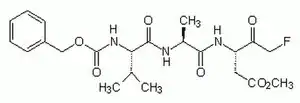 | Peptide modified |
| Mg^2+^ |  | Cation |
| Zn^2+^ |  | Cation |
| Ca^2+^ |  | Cation |
| Ca^2+^ |  | Cation |
| Antibodies |  | Protein |
| Antibodies |  | Protein |
| Antibodies |  | Protein |
